# Chinese Immigrant Women's Attitudes and Beliefs About Family Involvement in Women's Health and Healthcare: A Qualitative Study in Chicago's Chinatown

**DOI:** 10.1089/heq.2017.0062

**Published:** 2018-08-01

**Authors:** Melissa A. Simon, Laura S. Tom, Ivy Leung, Shaneah Taylor, Esther Wong, Dan P. Vicencio, XinQi Dong

**Affiliations:** ^1^ Department of Obstetrics and Gynecology, Northwestern University Feinberg School of Medicine, Chicago, Illinois.; ^2^ Robert H. Lurie Comprehensive Cancer Center of Northwestern University, Chicago, Illinois.; ^3^ Chinese American Service League, Chicago, Illinois.; ^4^ Mercy Hospital and Medical Center, Chicago, Illinois.; ^5^ Institute for Health, Health Care Policy and Aging Research, Rutgers, The State University of New Jersey, New Brunsiwck, New Jersey.

**Keywords:** immigrant health, family health, elderly, qualitative research

## Abstract

**Purpose:** Healthcare utilization and health-seeking behaviors of Chinese American immigrant women may be influenced by longstanding cultural perspectives of family roles and relationships. An understanding of Chinese immigrant women's perceptions of family social support in health and how these beliefs manifest in healthcare utilization and help-seeking behaviors is critical to the development of culturally appropriate health interventions. Focusing on a sample of Chinese women in Chicago's Chinatown, this qualitative study seeks to describe women's attitudes and beliefs about spouse and adult children's involvement in women's health and healthcare.

**Methods:** We conducted six focus groups among 56 Chinese-speaking adult women in Chicago's Chinatown between July and August 2014. Focus groups were transcribed, coded, and analyzed for emergent themes.

**Results:** Women reported that their adult children supported their health and healthcare utilization by helping them overcome language and transportation barriers, making and supporting decisions, and providing informational and instrumental support related to diet and nutrition. Women viewed these supports with mixed expectations of filial piety, alongside preferences to limit dependency and help-seeking because of concern and emotional distress regarding burdening adult children. Women's expectations of the spouse involvement in their healthcare were low and were shaped by avoidance of family conflict.

**Conclusion:** Findings inform opportunities for the development of culturally appropriate interventions to enhance Chinese immigrant women's health and healthcare. These include patient navigation/community health worker programs to promote self-management of healthcare and family-centered strategies for enhancing family social support structures and reducing family conflict.

## Introduction

Chinese Americans number over 4.5 million people and constitute the largest Asian subgroup in the United States.^1^ An estimated 76% of Chinese in the United States are foreign-born.^1,2^ Despite being widely viewed as better educated, more well off, and having better health outcomes than the general U.S. population, large pockets of the U.S. Chinese immigrant population belie these averages. This is particularly true of Chinatown ethnic enclaves, home to primarily low-income, working class, linguistically isolated Chinese immigrants. For example, in Chicago's Chinatown, nearly half of households have income less than $25,000, 37% of individuals have less than a high school education, and 57% do not speak English well.^3^

Health status of Chinese immigrants in the United States is more alarming than first impressions suggested in overall averages—especially considering the health disparities experienced by Chinese immigrant women. Compared with the general U.S. population, Chinese immigrant women in the United States experience disproportionate disease burden for several cancer sites (i.e., of the stomach, colon, liver), and chronic conditions such as hepatitis B and uncontrolled hypertension.^4–6^ Mental health issues, including psychological distress, depression, and suicidal ideation, are heightened among Chinese immigrant women compared with their male counterparts.^7,8^ Key to tackling these health disparities is to improve access to healthcare services, healthcare utilization, and health-related help-seeking behaviors among U.S. Chinese immigrant women.^5,6,9^ However, few studies have been conducted among this population examining factors associated with access, healthcare utilization, and health-related help-seeking behaviors. Barriers that have been identified from prior studies include those related to low health literacy, socioeconomic status, environmental effects, and healthcare provider/systems.^5,6,10^

Although the literature is growing and has illuminated contributing factors across multiple levels—individual, provider, and contextual—few studies have examined the role of family social support, or the processes by which family relationships promote healthcare utilization and health-seeking behaviors among Chinese immigrant women in the United States.^11–14^ This dearth of studies is surprising, given widely accepted cultural perspectives tracing back to Confucianism with regard to gender/family roles and relationships in caregiving, such as traditional three-generational households, women as caretakers of the family, and filial piety (obligation of children to respect and caregive for aging parents).^14–17^ But with evolving Chinese family structures, filial expectations, and practices of care provision,^7,13,18–20^ ongoing research is needed. Prior studies among U.S. Chinese immigrants have primarily documented expectations of family support for elder care and chronic medical conditions, yet few studies have focused specifically on the roles of the spouse and adult children in Chinese immigrant women's overall health and healthcare.^12,13,19,21,22^ Moreover, few studies have explored how Chinese immigrant women in the United States. conceptualize family support in women's health and healthcare.^23^ Largely unexplored are perceived constraints of family social support and how these beliefs manifest in women's healthcare utilization and health-seeking behaviors.

Thus, we report the results of a qualitative study on women's attitudes and beliefs about family involvement in women's health and healthcare. This study utilized focus groups conducted among Chinese immigrant women in Chicago's Chinatown, a densely populated area that is home to predominantly low-income, working class Chinese immigrants with low healthcare utilization.^3^ Specifically, we set out to explore women's perceptions of the roles of their spouse and adult children and perceived constraints to their involvement. We then discuss how research findings from our study among women in Chicago's Chinatown can inform the development of culturally appropriate health interventions for this specific population, with potential relevance to Chinese immigrant populations across the United States.

## Methods

Qualitative methods, such as focus groups, are valuable for collecting meaning-centered, contextually-based data.^24^ Focus groups soliciting Chinese immigrant women's opinions about women's health and experiences with medical care in the United States were conducted as part of formative work for a parent study, an intervention implementation study of cancer patient navigation among adult women (age 21+) residing in Chicago's Chinatown. Guided by our previous work and the existing body of literature on factors influencing Chinese and other minority and immigrant women's health practices,^23,25,26^ we constructed focus group questions to elicit women's attitudes and beliefs about involvement of their immediate family members (i.e., spouse, children) in women's health and healthcare, as rooted in cultural beliefs, values, and life experiences.^27^ Questions were translated into Chinese and arranged in three topic areas within the semi-structured moderator's guide: (1) perceived family members' (spouse and children's) involvement in medical care and health; (2) perceived family members' opinions on health-related matters; and (3) women's preferences and perceived constraints to family involvement. See Table 1 for sample questions. Other questions within the moderator's guide as part of the parent study pertained to interactions with healthcare providers, and health promotion and wellness messaging—the findings of which we will report separately. The Northwestern University Institutional Review Board approved all study procedures.

**Table 1.** **Qualitative Instrument**

Take a minute to think about your [family member^a^]…
What are some things that your [family member] does that helps you stay healthy?
What are some things that your [family member] does that is bad for your health?
How is your [family member] involved in your medical care?
Do you ask your [family member] for advice on matters related to health? What are some examples?
What do your [family member] think about…
Helping you make medical decisions?
You having a male doctor?
Accompanying you to the doctor?
You seeing a doctor in the United States vs. going back to your home country to see a doctor?
You getting a clinical breast exam and a pelvic exam?
You getting cancer screening tests?
Are there health issues where your [family member's] opinion differs from yours? What are some examples?
What do you think causes their opinion to be different from yours?
What usually happens when your [family member's] health opinions differ from yours? How are decisions made when this happens?
How involved do you want your [family member] to be when it comes to your health and healthcare?
What specifically would you want your [family member] to do for your health and healthcare that they are currently not doing now?
For those of you who want your [family member] to be more involved in your health and healthcare, what do you think kept them from being more involved in the past?
For those of you who don't want your [family member] to be more involved, why do you feel this way?

^a^ Family member: spouse, children.

### Study setting

Chicago's Chinatown community is home to over 42,000 Chinese immigrants and their descendants from mainland China, Hong Kong, and Taiwan.^3^ Among all Chinese living in Chicago and its surrounding suburbs, those with the lowest socioeconomic position reside in Chinatown, a densely populated commercial and residential area located in the South Side of Chicago. Together, the commercial core and adjacent residential neighborhoods make up Chicago's “Greater Chinatown area.” Unlike many other urban Chinatowns across the United States that have undergone gentrification, Chicago's Chinatown remains a community composed of lower-income, working-class Chinese immigrant families.^28^

### Recruitment and data collection

A convenience sample of Chinese women was recruited through word-of-mouth and flyers distributed at Chicago Chinatown community organizations that solicited participants for a discussion about women's health. As focus groups were conducted as formative work to inform design and implementation of a patient navigation program for adults in Chicago's Chinatown, eligible women for the focus groups were as follows: (1) self-identified Chinese; (2) spoke Cantonese or Mandarin; (3) age 21 and older; and (4) resided in Chicago's Chinatown. Study staff screened individuals for eligibility by phone and scheduled participants to groups according to Chinese dialect (Cantonese or Mandarin). We targeted focus group size of 8–12 participants each to allow for a wide range of experiences—inclusive of those who may have more or less to share than others.^29^ Between July and August 2014, a team of three bilingual research assistants (native Cantonese or Mandarin speakers) conducted three focus groups in Cantonese and three in Mandarin, maximizing study resources. Focus groups took place in a private room at a restaurant within Chinatown. Written informed consent was obtained before each focus group session, followed by administration of an anonymous sociodemographic questionnaire that included marital status and family composition. Discussion then proceeded using the moderator's guide. Each focus group had one dialect-concordant moderator and one note-taker from the team of three research assistants who were trained by the investigative team to facilitate focus groups according to study protocol and the semi-structured moderator's guide. The note-takers tracked participant comments to facilitate subsequent transcriptions and made observational field notes of nonverbal cues. Focus groups were audio-recorded and lasted ∼90 min, for which participants received a $15 gift card.

### Data analysis

Focus group recordings were transcribed verbatim by the bilingual focus group moderator team and translated into English by a certified translator. Translations were reviewed against the transcripts by the bilingual focus group moderator and note-taker team for accuracy. Analysis was a multistage process, using steps outlined by Strauss and Corbin.^30^ For thematic content analysis, members of the research team independently reviewed transcripts to identify initial coding schemes to add to the predefined themes derived from the moderator's guide. Coding schemes were compared and discussed until consensus was reached about a higher-level coding scheme. Two team members (S.T. and I.L.) then independently coded all transcripts using ATLAS.ti software. Discrepancies in coding were resolved through discussion. Finally, codes were classified into broader categories during examination for emerging themes. When appropriate, forms of social support were categorized by type (i.e., instrumental, informational, appraisal, and emotional). As is standard practice in qualitative research, we used qualitative descriptions and exemplar quotes to convey the breadth and strength of agreement with a statement, rather than quantifying responses.^29^

## Results

Sociodemographic characteristics of the 56 focus group participants are presented in Table 2. Over two-thirds (68%) were age 50 and over, and 27% had less than a high school education. All participants were born outside of the United States, with 59% having resided in the United States for at least 10 years and 27% for over 20 years. Most participants (62.5%) were married and 57.1% had children (age of children was not collected); only 10 of the 56 participants did not have a living spouse or child (Table 3). Most participants (66%) had public health insurance but 19% were uninsured. Key qualitative findings are subsequently described, organized based on themes. Quotations representative of themes regarding roles of adult children are reported in Table 4 and themes regarding roles of the spouse are reported in Table 5; age of each participant quoted is also detailed in Tables 4 and 5.

**Table 2.** **Sociodemographic Characteristics of Focus Group Participants (*n*=56)**

	*n*	%
Age		
21–39	4	7.1
40–49	13	23.2
50–59	6	10.7
60–69	21	37.5
70+	11	19.6
Missing	1	1.8
Educational attainment		
<High school	15	26.8
High school graduate	22	39.3
Some college (1–3 years)	7	12.5
College graduate or higher	9	16.1
Years in the United States		
0–5	10	17.9
6–10	12	21.4
11–15	11	19.6
16–20	7	12.5
20<	15	26.8
Marital status		
Married	35	62.5
Divorced	3	5.4
Widowed	12	21.4
Separated	1	1.8
Single, never been married	3	5.4
Missing	2	3.6
Composition of living family		
Spouse	35	62.5
Son(s)/daughter(s)	31	57.1
Son/daughter-in-law	5	8.9
Grandchildren	5	8.9
Sibling(s)	3	5.4
Other(s)	2	3.6
No living spouse or children	10	17.9
Health insurance coverage		
Uninsured	9	16.1
Public insurance (Medicare, Medicaid)	37	66.1
Private insurance	5	8.9
Other insurance	5	8.9
Have a primary care provider		
Yes	37	66.1
No	17	30.4
Don't know	2	3.6
Preferred language		
Cantonese	27	48.2
Mandarin	29	51.8

**Table 3.** **Composition of a Participant's Immediate Living Family Members, by Age**

Age group	*n* (%)	Spouse	Son	Daughter	Son/daughter-in-law
21–39	4 (7.1)	0	0	0	0
40–49	13 (23.2)	11	7	6	0
50–59	6 (10.7)	5	1	3	0
60–69	21 (37.5)	14	7	9	4
70+	11 (19.6)	4	1	4	1
Missing	1 (1.8)	-	-	-	-
Total	56	34	16	22	5

**Table 4.** **Beliefs and Preferences Regarding Roles of Adult Children in Women's Healthcare**

Themes		Representative statements
Social support (including instrumental, informational, appraisal, and emotional)	Language	“If there is really no way about it, say if there is a language barrier with the doctor over there, can't understand, then [my son] has to get involved.” (age: 66)
		“I only require help because my English isn't good.” (age: 69)
		“We don't understand English. Everything is left to him.” (age: 49)
	Transportation	“If I have anything, it's always [my son] who brings me to look for a doctor.” (age: 53)
		“The place is very, very far away. Sometimes I have to ask my daughter to drive me there. But they have to work. So I think it is very inconvenient.” (age: 65)
	Decision making	“My son will agree with my [medical treatment] decision. He would not say another word.” (age: 69)
		“[My son] agrees on doing [cancer screening tests] as soon as possible.” (age: 66)
		“I'll see whichever doctor [my son] decides. I totally don't know, it's always him who is searching.” (age: 53)
	Emotional support	“My youngest son, he studied medicine […] I was too afraid to go in when I saw the word “cancer” something “center,” I was too afraid to go in. I was shivering. I said I don't have that disease. My youngest son said you should go in, you should go there first. You don't need to be afraid. [My son] said many things can be cured. If discovered early, everything can be cured. He said there is no need to worry.” (age: 57)
	Diet	“My son is very good. He and my daughter-in-law follow a very healthy diet. They always say do not eat food that is too salty and too greasy.” (age: 70)
		“[My son] drives for grocery shopping on Saturdays and sometimes he reminds me not to eat too much salt because I have high blood pressure.” (age: 71)
Filial piety	Impact on health	“A harmonious family, then the body will be healthy.” (age: 60)
		“Unfilial children would be bad for the health. It's the only point. As long as he is filial. One is filial and one is not, one is beneficial for health, and one is not beneficial for health.” (age: 66)
	Expectations	“When we get old, we have to rely on our children.” (age: 71)
		“Nowadays, there are more of us old folks who care about you youngsters, and less of youngsters who care about us old folks!” (age: 60)
		“The dilemma is this. That is [he is] not well off. You have to spend a lot, a lot of money to seek treatment. I think even if he is filial, he is also unable to show that he is filial. There's no way.” (age: 72)
	Receipt	“When I decide to see the doctor, to have the operation, most likely he'll rush back on the day of the operation. Or my daughter will rush back, then accompany me for a week. Once I get better, then they would leave again. Filial is filial.” (age: 69)
		“No one takes care of me. I go to the doctor myself, my daughter is now married, what can I do?” (age: 60)
Limiting involvement	Time burden	“You have to take off for the whole day to accompany him/her to see the doctor, and sometimes a whole day is not enough. Or like her, had to go to Cook County for two days. These two days, you heard she said her son-in-law accompanied her, her daughter accompanied her, her husband accompanied her and her brother accompanied her, wasting so many human resources. See, this is the waste of U.S., waste of resources, waste of human resources, and this is really not good.” (age: 49)
		“You just shouldn't ask them for help all the time, they have to work.” (age: 69)
	Autonomy	“When it comes to their sons and daughter for old people […] if you have to get him to do it, even if he has no means of doing it, he'll still be able to do it. But it seems that for us, we try to do as much as possible by ourselves. […] If after letting them know, and there's nothing they can do to help you, it's not that they won't be glad, or unhappy, but they will be burdened, right? But we have our own thinking, right? So that's why we say to do as much as possible by ourselves. Only tell them about it when there is really no way around it.” (age:72)
		“I try my best to not give [my son] troubles. But as long as he knows that I have an illness, he'd be following throughout the entire process. During those times, the children have work as well. When you are sick, he'll definitely take leave […] So I try my best to not get him involved. If I am able to resolve it myself, then I resolve it myself.” (age: 66)
	Hiding illness	“Will tell them if there is the need to trouble them.” (age: 53)
		“I don't dare to tell my son whenever I'm sick. Once I let it out, he'll be calling every single day.” (age: 66)
	Emotional distress	“I'm worried that the person who takes you will hate it. He has work, he used this time to accompany you. If you spend a day, he'll accompany you for a day, if you spend half a day, he'll accompany you for half a day. Just this thing is bad. Auntie's high blood pressure was caused by anger. Not that she was mad at wasting her own time, but mad at wasting her son and daughter's time. A hard to come by day off was wasted by accompanying you for a day.” (age: 49)
		“My own time doesn't really matter. The main thing that I'm concerned of, is that the children don't have that much time. So every time I'm there to take my blood pressure, it'll go up.” (age: 66)

**Table 5.** **Beliefs and Preferences Regarding Roles of Spouse in Women's Healthcare**

Themes		Representative Statements
Social support (including instrumental, informational, appraisal, and emotional)	Transportation	“Bringing you there is good enough already.” (age 72)
		“Since I am sick a lot I have more illnesses […] he goes with me for doctor appointments. He is like a bodyguard to me.” (age 69)
	Help-seeking	“He'll support [me] going to take a look for any illness.” (age 49)
		“He thinks that I visit the doctors even with little problems […] For example, I have very bad hay fever that makes me unable to sleep. He thinks I can take some hay fever pills instead of treating it so seriously and seeing the doctor. We have different views on things, sometimes. (age 69)
	Decision making	“My husband insists on buying the best insurance plans.” (age 57)
		“When I go to take an inspection at a gynecologist, I always look for a female doctor. My husband might be a little conservative as well. If he sees that it is a male doctor, he wouldn't let me go.” (age 71)
		“I did tell him that I saw a male doctor today, he didn't really feel anything about it. A doctor is a doctor.” (age 49)
	Diet	“I am very lucky to have married my husband. I go to work and he comes home to cook.” (age 46)
		“He would remind me things like what to eat and what not to eat.” (age 69)
	Household chores	“He took good care (of me) in every aspect. Household, meals, he took care of the heavy liftings. I seldom did the heavy liftings and I let him take care of the meals. […] If you are sick, he would roll up his sleeves and help you.” (age 69)
		“He did almost everything physically and work-wise.” (age 69)
Limiting involvement	Perceived helpfulness	“My husband can tag along, and he can accompany. But there are things that he won't be able to help with.” (age 67)
		“Accompanying is enough, he won't be able to help you on the other things.” (age 49)
		“If you want the husband to help, then you should have gotten a doctor for a husband.” (age 49)
	Work priorities	“I am the one helping him, he wouldn't seek treatment even when he is sick. […] So needless to say, if I was sick, he wouldn't be helping me. I have to take the initiative to go by myself. If not, my children, son and daughter, would take me. So my husband will always be like this for his lifetime, only knows about work, never knowing to care about himself and his family.” (age 66)
		“He won't do it if he doesn't have time, would do when he's free. (He) doesn't have time because he has to work.” (age 46)
	Conflict avoidance	“If he were to be sarcastic, your illness will only become worse, it's like that, isn't it? ‘Don't bother seeing, it's only a little illness.’ This would worsen your illness. Bad mood would cause a person to feel uncomfortable.” (age 49)
		“When men reach the age of 70 years old, I discovered that their temper starts to become bad. When (he) talks, he wouldn't reason with you. He does his own talking. All I can do is keep quiet, right?” (age 69)
	Perceived lack of concern	“He is the type of person who is not afraid of anything, doesn't care much about anything.” (age 69)
		“He doesn't really care about [health] […] Now I am more mature, I am older, and he cares about me less. He doesn't really get involved or give opinions.” (age 69)

### Beliefs and preferences regarding roles of adult children in women's health and healthcare

Analysis revealed that across focus groups, women's perceptions of their adult children's involvement in healthcare revolved around domains of social support, filial piety, and limitations to involvement.

#### Social support

Various forms of social support, including instrumental, informational, appraisal, and emotional support from adult children were mentioned during focus groups to different extents. Women primarily described instrumental support from adult children, especially reliance on adult children in overcoming language and transportation barriers. As one participant described, language was the only reason for needing support from her son: “I only require help because my English isn't good.” Another lamented the distance to health clinics: “The place is very, very far away. Sometimes I have to ask my daughter to drive me there.” Similarly, driving them grocery shopping was mentioned by women as a notable aspect of adult children's involvement in their health and healthcare. Women's description of informational and appraisal support came in the form of adult children's involvement in decision making about healthcare access and utilization. When it came to selection of healthcare providers, some women described taking advice from adult children, noting for example, “I'll see whichever doctor [my son] decides.” On the other hand, others described their adult children's role in just affirming healthcare decisions: “my son will agree with my [medical treatment] decision. He would not say another word.” Women also mentioned nutritional advice as notable aspects of adult children's involvement in their health and healthcare. Emotional support was only directly mentioned in the context of serious healthcare concerns. For example, one woman, who underwent tests for leukemia, recounted: “I was too afraid to go in [to the cancer center] […] My youngest son said you should go in, you should go there first. You don't need to be afraid.”

#### Filial piety

Women's attitudes and preferences about family involvement in their health and healthcare were informed by their beliefs regarding filial piety. While a few women put forth clear statements such as “when we get old, we have to rely on our children” and “unfilial children would be bad for health,” many women noted the complexities regarding expectations and receipt of filial piety. Expectations were colored by financial realities and competing priorities. One woman stated of her son: “[He is] not well off […] I think even if he is filial, he is unable to show that he is filial.” Another woman remarked about her limited expectations for her daughter: “My daughter is now married, what can I do?”

#### Limiting involvement

Adult children's roles were particularly constrained by social/cultural norms to limit family burden. Women conveyed concerns and emotional distress about burdening their children. For example, one woman spoke of her distress for having her adult children accompanying her medical visits, by describing: “Auntie's high blood pressure was caused by anger. Not that she was mad at wasting her own time, but mad at wasting her son and daughter's time. A hard to come by day off was wasted by accompanying you for a day.” Burden was felt in relation to their children's job commitments: “you just shouldn't ask them for help all the time, they have to work.” The burden corresponded to preference among women to be independent about health-related matters. As one woman remarked: “I try my best to not get [my son] involved. If I am able to resolve it myself, then I resolve it myself.” A prevalent theme was limiting health-related communications with adult children to spare them from concern. For example, one woman shared: “I don't dare to tell my son whenever I am sick. Once I let it out, he'll be calling every single day.” Women did not mention different beliefs and preferences in limiting involvement of sons compared with daughters.

### Beliefs and preferences regarding spousal roles in women's health and healthcare

With respect to women's perceptions of spousal involvement in their health and healthcare, analyses revealed that beliefs also centered around domains of social support and limitations to involvement.

#### Social support

Similar to perceived social support from adult children, when women were asked about their health and healthcare and the involvement of their spouse, women's descriptions of spousal instrumental, informational, and appraisal support varied, especially on topics related to spouse encouragement to seek care when ill, transportation support, and decision-making support. Some women perceived no spousal involvement in their healthcare. Some noted instrumental support in the form of their spouse providing transportation. With respect to informational and appraisal type social support, some commented that their spouse would tell them to go see the doctor when ill, but many others recounted their spouse downplaying their concerns. Women also had disparate views on their spouse's support on choice of health care providers, ranging from, “If he sees that it is a male doctor, he wouldn't let me go,” to “I did tell him that I saw a male doctor today, he didn't really feel anything about it.”

Rather than provision of direct support in relation to women's health and healthcare, many women expressed that their spouse's key contribution was involvement in food preparation and household chores. Regarding food preparation and nutrition, women described instrumental and informational support from a spouse cooking meals and advising on “what to eat and what not to eat.” Other examples of perceived spousal instrumental support for health involved helping with housework. One woman observed, “If you are sick, he would roll up his sleeves and help you.”

#### Limiting involvement

Constraints to existing spouse involvement and preferences regarding future involvement included perceptions of spouse's competing priorities, such as work and being the primary income earner. For example, one woman commented about her husband's limited involvement due to work priorities, “He doesn't have time because he has to work,” while another woman also lamented: “I am the one helping him, he wouldn't seek treatment even when he is sick […] So needless to say, if I was sick, he wouldn't be helping me. I have to take the initiative to go by myself […] My husband will always be like this for his lifetime, only knows about work, never knowing to care about himself and his family.” Related to cultural norms of spouses having competing priorities such as work, some women perceived that their spouse was unconcerned about health. As one woman succinctly stated, “He doesn't really care about [health].” When asked about their preferences for spousal involvement, some women noted that even if their spouse did concern themselves with matters of healthcare and had available time—transportation and accompaniment would be the extent of healthcare involvement due to circumstances of language and unfamiliarity with medicine. As one woman described, due to spouse's language and medical knowledge barriers, “He can accompany. But there are things that he won't be able to help with.”

Cultural norms related to avoiding family conflict may be another perceived constraint to spousal roles in women's health and healthcare. For example, one woman commented on her behavior in reaction to her husband's temper, stating: “When [he] talks, he wouldn't reason with you. He does his own talking. All I can do is keep quiet, right?” Moreover, there were perceived health consequences from family conflict arising from discussion of health issues. For example, thinking about preferences for family involvement, one woman explained her reluctance to discuss health issues with her husband because “If he were to be sarcastic, your illness will only become worse.”

## Discussion

Understanding Chinese immigrant women's perceptions of family social support in health and how these beliefs manifest in healthcare utilization and help-seeking behaviors is critical to the development of effective, culturally appropriate health interventions. This study is among the first to describe women's attitudes and beliefs about spouse and adult children's involvement in women's health and healthcare in the United States. Overall, women reported that their adult children supported their health and healthcare utilization by helping them overcome language and transportation barriers, making and supporting decisions, and providing support related to diet and nutrition. Women viewed these supports with mixed expectations of filial piety, alongside preferences to limit dependency and help-seeking because of concern and emotional distress over burdening adult children. Women's expectations of the spouse involvement in their healthcare were low and shaped by avoidance of family conflict. These findings add nuance to what is currently known about family support in U.S. Chinese immigrant women's health and healthcare.

Not surprisingly, language and transportation were the most expressed instrumental support provided by adult children. Unlike more rigid notions of filial piety, whereby adult children must provide care for elderly parents or else cause shame,^31^ our findings suggest that Chinese immigrant women accepted that adult children offered what available support they could. Much of the literature attributed decline in filial piety to migration and exposure to Western values,^13,25^ our findings also speak of systemic issues regarding financial hardship, work environments, and economic policies that limit time and resources for caregiving. We found that one aspect of filial piety—emotional support—was largely missing, and only mentioned in the context of serious illness. Prior studies suggest that emotional support may be more important than instrumental (e.g., financial and practical) support,^25,32^ so perceived dearth of emotional support may have troubling implications.

A key concern among women was that their reliance on adult children for health-related matters interfered with children's job commitments. Feelings of burdening their children translated to emotional distress, paralleling findings from a study of Chinese cancer patients that identified anxiety of impacting the family as the most difficult aspect of living with cancer.^21^ In response to complex feelings about burden, we found that women triaged health-related communications with their adult children and minimalized help-seeking. Unfortunately, limited information may present a challenge for adult children looking to optimize involvement in their aging parents' healthcare.

Our results suggest that expectations of spouses' roles in healthcare may be constrained by women's preferences to avoid family conflict. Gender roles and norms stemming from Confucian philosophy may make it challenging for women to directly express their needs over their spouse's.^13^ For example, studies of diabetes management among Chinese Americans have suggested that women's responsibility for their family's emotional well-being may result in women having less emotional support to address individual needs.^22,33^ While times of illness have been found to increase spousal support in a study of Chinese American immigrants,^22^ our findings suggest that by and large, Chinese immigrant women do not feel emotionally supported by their spouse with respect to health-related matters.

Before we discuss implications of study findings, key study limitations should be noted. First, the data represent a convenience sample of women residing in Chicago's Chinatown. As all participants were foreign-born, most have resided in the United States for over 10 years, and most were recruited from community settings, we recommend caution in generalizing study findings on U.S.-born Chinese Americans, and more recent or socially isolated immigrants. Another limitation is with regard to our focus group format. As discussion topics may be stigmatized or perceived as sensitive, women in group settings may not wish to describe negative family relationships. Thus, we expect that family healthcare involvement may be overstated, and that actual need among Chinese immigrant women may be greater than reported here. Moreover, as we focused on women's perceptions, we did not obtain direct accounts from adult children or spouses to triangulate differences in perceived healthcare involvement. Nor did focus groups topics dive more deeply into healthcare decision-making processes or differences in expectations of sons versus daughters to place findings on attitudes, beliefs, and preferences for social support into greater context. Despite these limitations, our study findings provide insights for development of culturally tailored interventions, including in areas of patient navigation, family-centered caregiving support, and mental health.

To enable women to manage their own healthcare, patient navigators—who make appointment reminder calls; provide informational, logistical, and emotional support; provide interpreter services; and refer patients to community resources—may be crucial for promoting women's health in Chinese immigrant communities. Patient navigation is not a new strategy in the United States and research has established its efficacy among non-Asian limited English proficient populations.^34–36^ However, our study finding that women prefer to limit their dependency on family members for healthcare needs lends support to the development of navigation programs tailored for Chinese immigrant communities, with particular emphasis toward helping women gain independence in healthcare and information-seeking. Indeed, patient navigation programs often struggle with what happens after the proverbial cord is cut.

Another clear need identified in our findings is for family-centered interventions to enhance family social support structures for caregiving. While family-centered interventions have previously been proposed and utilized across health topics, such as Alzheimer's disease, dementia, stroke, and other chronic conditions, most of these studies focus on highlighting caregiver burden and coping strategies such as stress reduction.^21,37–40^ Our findings regarding a perceived dearth of emotional support from family members suggest that strategies and resources are needed to enhance family delivery of emotional support. Moreover, our findings of time burden to adult children and conflicting work priorities suggest that resources should be accessible on-demand. With the growing ubiquity of web and mobile apps, technology will likely play larger roles in women's health-related social support,^41^ so enhancing readily accessible online resources and tools may be promising avenues for intervention development. In addition, our findings suggest the need for intervention strategies to help spouses normalize assistance and support to wives with respect to health and healthcare seeking behaviors. As an example, couple-based interventions have demonstrated promise in improving communications in cancer caregiving^42^ and management of chronic illness,^43^ although these intervention strategies have not been tested among Chinese immigrant populations.

Women valuing harmony with their spouse over expression of needs, women experiencing emotional distress over burdening adult children, and women adopting communication patterns that limit help-seeking all speak on the need for greater attention to mental health among Chinese immigrant women in the United States. Interventions for strengthening relationships and communication, and managing family conflict may involve family psychotherapy, counseling, and family mediation approaches, perhaps extensions of caregiving interventions to bridge differences in caregiving expectations and provision.^44^ Previously, group interventions to build skills for dealing with family and social dilemmas in the context of diabetes management have resonated well with both genders.^22^ Mental health providers also need to recognize the potential impact of family cultural conflict.^45^ Psychotherapy can be culturally tailored to help Chinese immigrant women cope with their anxiety of burdening their families, and conflicts arising at the intersection of cultural norms, sense of self, and expression of needs.^46^ Ultimately, access to mental health services is pivotal for Chinese immigrant women, especially if emotional support is unavailable from the spouse or adult children.

## Conclusion

This qualitative study exploring Chinese immigrant women's perceptions of family social support found reliance on adult children for some forms of instrumental support. But women festered complex concerns of burdening adult children and preferences to limit dependency on health-related matters. Women's expectations of spousal involvement in healthcare were low. Findings present opportunities for the development of culturally appropriate interventions to enhance Chinese immigrant women's health and healthcare.
